# Atomic ring invariant and Modified CANON extended connectivity algorithm for symmetry perception in molecular graphs and rigorous canonicalization of SMILES

**DOI:** 10.1186/s13321-020-00453-4

**Published:** 2020-08-20

**Authors:** Dmytro G. Krotko

**Affiliations:** 1 Enamine Ltd, 78 Chervonotkatska Street, Kyiv, 02094 Ukraine; 2 Chemspace LLC, 85 Chervonotkatska Street, Kyiv, 02094 Ukraine

**Keywords:** Molecular graphs, Symmetry perception, SMILES, Canonicalization

## Abstract

We propose new invariant (the product of the corresponding primes for the ring size of each bond of an atom) as a simple unambiguous ring invariant of an atom that allows distinguishing symmetry classes in the highly symmetrical molecular graphs using traditional local and distance atom invariants. Also, we propose modifications of Weininger’s CANON algorithm to avoid its ambiguities (swapping and leveling ranks, incorrect determination of symmetry classes in non-aromatic annulenes, arbitrary selection of atom for breaking ties). The atomic ring invariant and the Modified CANON algorithm allow us to create a rigorous procedure for the generation of canonical SMILES which can be used for accurate and fast structural searching in large chemical databases.

## Introduction

The perception of the symmetry of atoms in molecular graphs is an important problem for chemoinformatics. The systems for the synthesis design use the constitutional symmetry information to generate and evaluate the reaction pathways [1]. The constitutional symmetry is important for the interpretation of NMR and ESR spectra. Computer-assisted structure elucidation systems also use this information [1]. The correct determination of the symmetry classes for the atoms in a molecular graph is a basis for finding a canonical ordering of the atoms in a molecule, which in turn is necessary for generating a unique representation of the molecule [1]. This approach is used in the canonicalization algorithms for the linear representations of the molecular graphs like SMILES [2–5] and InChI [4, 6]. The canonical linear notations of the molecular graphs are widely used in the contemporary chemical databases since they allow to compare chemical structures as plain strings. These strings could be lexicographically ordered, which allows using very fast binary searching in ultra-large databases of canonicalized linear representations of molecular graphs [1]. The accuracy and speed of search in millions of known and billions of virtual compounds for drug discovery relays on the correctness and the effectiveness of these algorithms.

**Fig.1:**
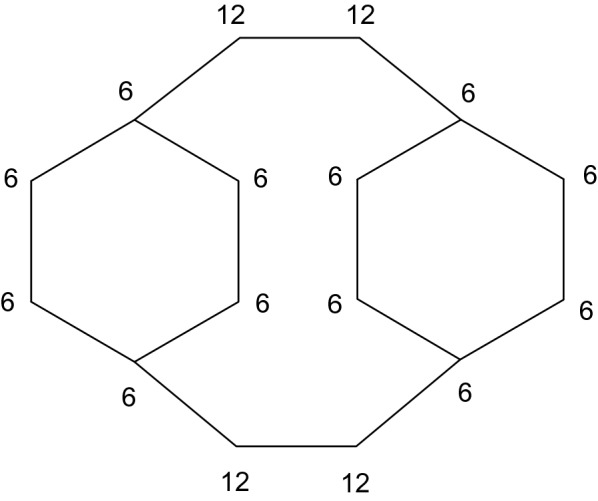
‘Pathological’ graph with the sizes of the smallest rings for the atoms

The first algorithm for the symmetry perception of the atoms in the molecular graphs was developed in 1965 by Morgan [7] for Chemical Abstracts Service. In the first phase of this algorithm, an initial value is assigned to each atom based on its local properties like degree, atomic number, and bond type. In a second so-called relaxation step, these values are iteratively refined by summing up of the current values of the immediate neighbors of each atom and assigning this new value to the atom. The algorithm is terminated when a count of different values ceases to increase from the previous step of the algorithm. This algorithm is a classical realization of the extended connectivity algorithm and works pretty well for most of the typical chemical structures. But in 1975 Randić has pointed out [8] that issues exist with this algorithm: the same extended connectivity values can be assigned for nonequivalent atoms in some molecular graphs. Some of these issues are related to the fact that the sum of integers is not unambiguous function and addition various integers can give the same sum. This problem could be solved by a method described by Weininger et al. [3] in 1989 by replacing summing at the refining step of the algorithm by the product of corresponding primes as an unambiguous function that can be easily shown from the prime factorization theorem. But the method of the corresponding primes is not universal: for some ‘pathological’ molecular graphs (one of them shown in Fig. 1) none of the extended connectivity algorithms, whose initial values assigned to each atom based on the local properties of atom only, can resolve all symmetry classes of these highly symmetrical molecular graphs [9]. This fact led lvanciuc to the statement [1] that only algorithms with an explicit determination of the graph automorphisms (an isomorphism of a graph with itself is called an automorphism) can accurately determine all symmetry classes in complex molecular graphs. Such algorithms, like Shelley-Munk algorithm [10], the HOC (Hierarchically Ordered Extended Connectivities) algorithm [11], McKay algorithm [12] (which is implemented in open-source library nauty) and Faulon et al. [13, 14] canonical signatures algorithm, are quite complex and computationally intensive since they use some sorts of the path enumerations and the label permutations. The simplified and adapted for the molecular graphs version of McKay algorithm (nauty) is implemented in InChI canonicalization algorithm [6]. Another approach is shown by Schneider, Sayle, and Landrum [5] in 2015 and is implemented in an open-source RDKit library. They use some nonlocal invariants of the atoms (the chirality invariant and the special high-symmetry invariant) for resolving the symmetry classes in the chiral structures and the highly symmetrical molecular graphs by their version of the extended connectivity algorithm. Using these invariants allows obtaining an accurate count of the symmetry classes in the chiral structures and ‘pathological’ molecular graphs, respectively. But I would like to propose another (from my point of view, more obvious for the chemists) nonlocal invariants of the atoms for the same purposes.

The mathematical theory of canonical coding of the graphs with application to the molecular graphs is outlined in the review by Ivanciuc [1]. For further discussion, we have to give the major definitions and facts from this review. A code of the labeled graph is a string obtained from graph by a set of rules. The code is a complete representation of graph because the labeled graph can be reconstructed from code. The code is not a structural invariant, because different labelings of graph usually give different codes. An important property of codes is that the lexicographical relation between two strings induces an order of the codes. A rigorous method to derive the canonical code and automorphism partitioning of the graph with N vertices is to construct all N! permutations, to generate their codes and compare them to extract the one-to-one correspondence. The permutation labelings corresponding to the canonical code are identified by a lexicographical comparison of the N! codes, followed by the selection of the maximal (or minimal) code. From the above definition it is clear that for a given molecular graph the canonical code is unique. This property is used in graph isomorphism testing and in storage, retrieval and comparison of chemical compounds, because two molecular graphs with identical canonical codes represent the same chemical compound. The process of generation of the canonical code by investigating automorphism permutations is called canonical code generation by automorphism permutation (CCAP). Since for practical purposes N! permutations is a very large number, all coding algorithms use a heuristic approach to reducing (in a deterministic way, which does not depend on labeling of the molecular graph) the number of permutation labelings that have to be investigated in order to detect the canonical labelings. A vertex graph invariant is any vertex property, computed on the basis of the graph structure, whose value does not depend on the graph labeling. Examples of vertex invariants are atomic number, the degree and distance sum. Any vertex invariant can be used to obtain a preliminary partition of the vertices from a molecular graph. Two vertices from different atom invariant classes (AIC) cannot be automorphic, while two vertices from the same AIC are not necessarily automorphic. Despite numerous efforts, no vertex graph invariant is known which is sufficient to establish the automorphism partitioning, because for certain graphs non-automorphic vertices are partitioned in the same class. The process of atom partitioning in AIC induced by a certain atomic invariant is called graph invariant atom partitioning (GIAP), and represents an important step in the generation of the canonical code. The use of the GIAP step is based on the property that two atoms with distinct values for the same invariant cannot be automorphic. On the other hand, the assumption that atoms in the same GIAP class are automorphic is not correct. To determine the canonical code, each AIC resulting from GIAP procedure is investigated to detect non-automorphic vertices by lexicographical comparison of all possible codes for this partitioning, followed by the selection of the maximal (or minimal) code.

Thus, a complete algorithm for canonical coding can be separated in 2 steps:

1. GIAP: compute a discriminant atom invariant and establish with it an initial atom partitioning.
2. CCAP: using the atom partitioning established in the first step identify canonical code by investigating all automorphism permutations.

Ivanciuc writes [1]: “Carhart pointed out that a rigorous canonical coding and constitutional symmetry perception algorithm must contain both GIAP and CCAP steps [9]. The same idea was formulated for the problem of graph isomorphism by Read and Corneil [15]. Even after these warnings, the illusion of obtaining a “better” and “faster” canonical coding algorithm by eliminating the step CCAP continued and one can still find in the literature papers that propose algorithms for the computation of vertex invariants, with the wrong assumption that vertices with identical values of the invariant are automorphic and belong to the same orbit. Even when such AIC algorithms give vertex partitionings that coincide with the automorphism partitioning for a certain set of graphs, this fact is not a demonstration that the algorithm can generate the automorphism partitioning for any graph. This type of “canonical coding” algorithm is incomplete, and its use in a chemical database, in synthesis design or structure elucidation systems is unreliable.”

Since both published by Weininger et al. [3] and by Schneider, Sayle, and Landrum [5] algorithms for canonicalizing of SMILES do not contain CCAP (canonical code generation by automorphism permutation) step, they are incomplete, i.e. for some molecular graphs they could give different canonical SMILES for the label permutations of one molecular graph. Unlike them, the InChI canonicalization algorithm [6] is complete. So our main goal is to create a complete but fast algorithm for SMILES canonicalization. To achieve it, we propose an efficient GIAP procedure, which in almost all cases of the molecular graphs gives the correct partition of the atoms of the molecular graph into symmetry classes, followed by the CCAP step that will guarantee the uniqueness of the canonical code for a given molecular graph.

## Methods

Herein, we discuss the use of the local invariants and propose nonlocal invariants and improvements of the refinement procedure that can resolve some ambiguities in the Weininger’s method of the corresponding primes [3]. Then, we describe a procedure to obtain a canonical absolute SMILES (with chirality). The material in this section is presented in the order in which the steps of our SMILES canonization algorithm are performed.

### Local invariants

Degree, atomic number, and bond type are used as the local invariants of atom in the original Morgan algorithm [7]. Weininger has proposed to use number of connections, number of non-hydrogen bonds (degree), atomic number, the sign of charge, absolute charge, and number of attached hydrogens as the local invariants [3]. For complete representation of the local invariants of atom, we can classify them as properties of the atom proper (atomic number – count of protons in atomic nuclei, atomic mass—the sum of counts of protons and neutrons in atomic nuclei, charge—count of protons in atomic nuclei minus count of electrons of the atom) and properties of the nearest neighborhood of the atom (degree—count of explicit connections, count of attached hydrogens, connectivity—total count of connections, valence—the sum of bond orders of all connections). For use as an initial rank of the atom, we must combine all local invariants in the atomic vector in some order. Weininger has pointed out [3] that a terminal atom is preferred to start of traversal of the molecular graph (if it is possible). For this reason, the degree of the atom must be at the start of a combined atomic vector (1 character). Next positions concatenated consecutively: atomic number (3 characters), count of attached hydrogens (1 character), the sign of charge (1 character: ‘0′—for no or positive charge, ‘1′—for a negative charge), absolute charge (1 character), connectivity (1 character), valence (1 character), atomic mass (3 characters, ‘000′ if unspecified). The atomic vector of the local invariants has 12 characters.

### Chirality invariant

In SMILES, a designation of chirality depends on the enumeration order of atoms, and from this reason, simple parity symbols ‘@’ and ‘@@’ cannot be used as invariant since its meaning changes with the transposition of the atoms. But we can determine the order of the atoms around a chiral center relative to the symmetry classes of its neighbors [16, 17]. To determine this order we should use the extended connectivity algorithm to the atomic invariants (local and ring and distance nonlocal described further) without chirality designation and obtain the symmetry classes for the non-chiral structure. For all atoms with no chiral parity, the chirality invariant is equal to 0 (by definition). If two or more neighbors of the atom with the chiral parity have the same symmetry class then the chirality invariant of this atom is equal to 0 too. If the order of the symmetry classes can be obtained by an even number of swaps from the direct ascending order of these numbers then the chirality invariant is equal to count of the sign ‘@’ in parity, and otherwise the chirality invariant is equal to 3—count of the sign ‘@’ in parity. Such a definition of the chirality invariant gives us the truly invariant property of the chiral atom that is independent of the order of atoms in SMILES because the symmetry classes of the atoms are independent of its order. This invariant can be concatenated to the atomic vector of each atom (1 character). After concatenation of the chirality invariant, we must recalculate all atomic ranks in molecular graph.

Thus, the complete atomic vector of each atom must contain 13 characters: 12 for the local invariants and 1 character for the chirality invariant. Since all characters of the atomic vector are digits then we can transform the atomic vector to number.

### Ring invariant of atom

If we use only traditional local invariants for each atom of the molecular graph from Fig. 1 we will obtain only two symmetry classes after the refinement procedure of the extended connectivity algorithm: one for the atoms with degree = 3 and another one for the atoms with degree = 2. But, there are three symmetry classes in this structure. How do we know that? Because eight atoms with degree = 2 belong to the 6-membered rings, but another four atoms with degree = 2 do not. This very simple observation gives us obvious nonlocal invariant for each ring atom—the ring size of the smallest ring to which this atom belongs (for non-ring atom this invariant is 0 by definition). This value is invariant, i.e. it does not depend on the order of the atoms in molecular graph. Now, for molecular graph from Fig. 1, for the eight atoms with degree = 2 this invariant is 6, and for other four atoms with degree = 2 this invariant is 12. Taking into account the local invariants of each atom we obtain three symmetry classes for the atoms of this molecular graph at once and the refinement procedure does not change this.

**Fig.2:**
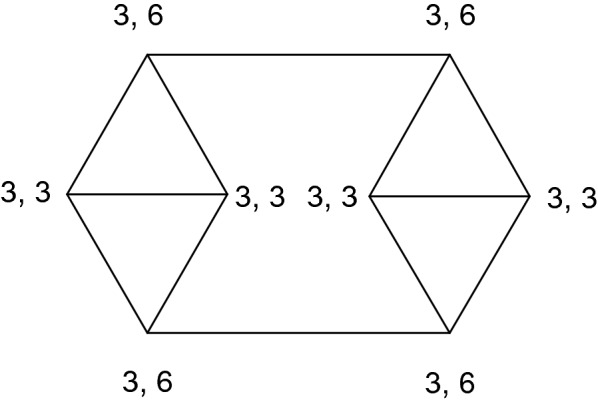
Complex graph with the minimal and maximal ring sizes of the bonds incident with all atoms

But if we look at the structure in Fig. 2, we see that this invariant is insufficient because all atoms belong to the 3-membered rings and therefore have the same ring size of the smallest ring to which this atom belongs.

To solve this issue, we can first calculate an invariant for each bond—the size of the smallest ring to which it belongs. We can use a breadth-first search (BFS) from the first atom incident with this bond to the second atom incident with this bond for obtaining the shortest non-trivial path (if such path does not exist then this is non-ring bond). After obtaining this ring invariant for each bond, we can choose for each atom minimal and maximal values of the ring sizes of the bonds incident with this atom. The minimal ring size of the bonds incident with the atom will be the ring size of the smallest ring to which this atom belongs, but maximal will be the maximum of the minimal ring sizes of the bonds incident with this atom. Both of these values will be invariants of each atom. For the molecular graph in Fig. 2, four atoms have these invariants equal to 3, but four other atoms have the minimal ring size 3, but the maximal ring size is 6. The minimal and maximal ring sizes of the bonds incident with the atom in combination with the local invariants are usually sufficient for distinguishing all symmetry classes of the atoms in ‘pathological’ molecular graphs. But to avoid any possible ambiguity we propose to use the product of the corresponding primes for the ring size of each bond of the atom as unambiguous ring invariant of the atom (contribution for a non-ring bond to the product will be 1). As an example, for the molecular graph in Fig. 2, this ring invariant will be 5*5*5 = 125 for the atoms with the maximum ring size of the bonds equals to 3 and 5*5*13 = 325 for the atoms with the maximum ring size of the bonds equals to 6 (since 5 is third prime and 13 is the sixth prime number). The atomic ring invariant will be unambiguous since reverse factorization of it gives us such invariants as count of the ring bonds of the atom and the minimal sizes of rings to which all bonds of the atom belong.

### Distance invariant of the atom

**Fig.3:**
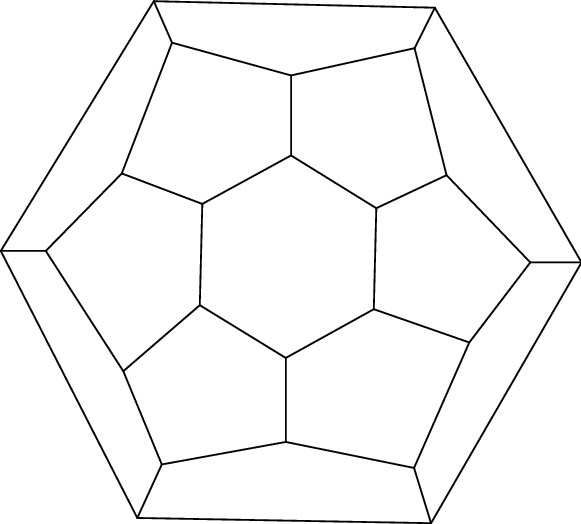
Example of the 24-fullerene molecular graph for which the ring invariant is not sufficient

Unfortunately, our local and ring invariants are insufficient for the molecular graphs of the fullerenes from the article of Laidboeur et al. [18]. For example, for the molecular graph in Fig. 3 (SMILES: C1(C2C3C4C15)C6C7C2C8C3C9C%10C4C%11C5C6C%12C%11C%10C%13C%12C7C8C9%13) our algorithm with the ring invariants of the atoms determines only one symmetry class for the atoms since all atoms of this molecular graph have degree equal to 3 and all bonds of this molecular graph belong to the smallest 5-membered rings while a complete search of the automorphisms gave us two symmetry class of the atoms [18].

To solve this problem, we tried to use various invariants of the atom which reflect the topological distance of each atom from all other atoms in the molecular graph. The first invariant is the distance sum—the sum of the topological distances from the current atom to all other atoms in the molecular graph [1]. This is a powerful atom invariant but it is hampered by the degeneracy (i.e., two or more topologically distinct vertices can have identical numerical values) [1]. Another proposed distance invariant of the atom is the number of the outermost occupied neighbor sphere (NOON) of an atom [19]. This is the minimum number of neighbor spheres necessary to accommodate all atoms of a molecule starting at that atom (in fact, the eccentricity of the vertex in the molecular graph—the maximum of the topological distances from the current atom to all other atoms in the molecular graph). For the determination of all topological distances from the current atom to all other atoms in the molecular graph we can use the breadth-first search (BFS) from the current atom. Our experiments showed that the weighted sum of the topological distances from the current atom to all other atoms in the molecular graph has the best separation ability: we define the distance invariant of the atom as the sum of the products of the count of the atoms which have the topological distance d from the current atom to N^d^, where N—some integer. For example, we can use N = 10, and thus the decimal digits of our distance invariant are the counts of the atoms in such topological distance from the current atom as the digit position number from the end of the decimal representation (this is the same as the invariant ‘number of neighbors per level’ in the reverse order of the digits [5]). The NOON of the atom will be equal to the overall count of the digits in the decimal representation of the distance invariant. For example, for the 24-fullerene 12 atoms have the distance invariant equal to 25,763, but 12 other atoms have the distance invariant equal to 35,663 (but all atoms have the NOON equal to 5). Such defined the distance invariant of an atom with the refinement step allows us to easily determine all symmetry classes of the atoms in the fullerenes from the article of Laidboeur et al. [18], but, unfortunately, all our local, ring and distance invariants of the atoms are not sufficient for the accurate determination of all symmetry classes for some complex graphs (see ‘Results and Discussion’ section). This fact is a limitation of our symmetry perception algorithm (as well as all known symmetry perception algorithms without the explicit determination of the graph automorphisms [1]), but since we use the canonical code generation by the automorphism permutation for obtaining of the canonical SMILES (see ‘Introduction’ section), even approximate values of the symmetry classes allow drastically reduce the count of the necessary permutations for the rigorous canonicalization of SMILES [1].

Now, we have the 13-digit atomic vector, the numeric ring invariant, and the numeric distance invariant for each atom. We can sort all atoms of the molecular graph by the numeric values of their atomic vector, ring, and distance invariants and assign the rank in the results of sorting as the initial rank of each atom for the refinement step of the extended connectivity algorithm.

### Refinement procedure

**Fig.4:**
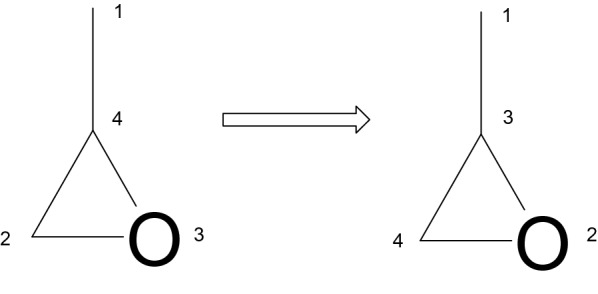
Example of the ranks rotation by the original CANON algorithm

**Fig.5:**
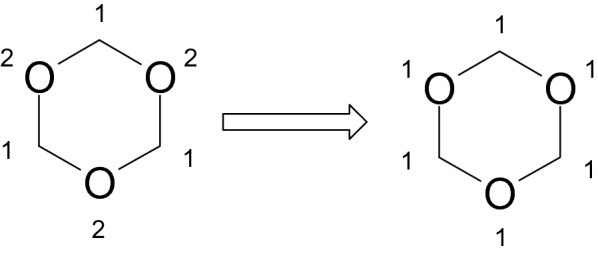
Example of the ranks leveling at the refinement step

After obtaining the initial ranks for all atoms we can rank them to obtain consecutive small integer ranks, and this manipulation does not change their relative order. Further manipulations, in general, correspond to the CANON algorithm described by Weininger et al. [3], but some changes in this algorithm are necessary. If you look at the simple structure in Fig. 4 then you see that after obtaining the initial ranks we have four symmetry classes. If we follow the original description of the CANON algorithm then after first iteration we obtain products of the corresponding primes for the atoms: CH_3_—7, CH—2*3*5 = 30, CH_2_—5*7 = 35, O—3*7 = 21. After ranking of these integers we obtain new ranks (Fig. 4):

As you can see, there is a rotation of the ranks in the ring. This is an infinite loop of the ranks rotations that cannot give us stable symmetry classes of the atoms in such type of structures. The first idea for solving this problem is to multiply the corresponding prime of the current rank of each atom with the product of the corresponding primes for the ranks of its neighbors. Employing this approach, the products for first iteration are: CH_3_—2*7 = 14, CH—7*2*3*5 = 210, CH_2_—3*5*7 = 105, O—5*3*7 = 105. After ranking we obtain that now O and CH_2_ have the same rank, although previously they had different ranks! This issue arises since the products of primes are the unambiguous function only if we do not consider the transposition of the number in the product. But in the 3-membered rings, this fact led to the leveling of the previously different ranks. A solution to this problem for the 3-membered rings is to multiply the square of the corresponding prime of the current rank of each atom with the product of the corresponding primes for the ranks of its neighbors. Using this approach, the products for the first iteration are: CH_3_—(2*2)*7 = 28, CH—(7*7)*2*3*5 = 1,470, CH_2_—(3*3)*5*7 = 315, O—(5*5)*3*7 = 525. After ranking: CH_3_—1, CH—4, CH_2_—2, O—3. As you can see, the ranks are stable after such calculation for this structure. It could be easily proved that such a method of obtaining new ranks from previous ones has never led to leveling or swapping the previously different ranks in the 3-membered rings. But for structure in Fig. 5, squaring the corresponding prime of the current rank does not help us: after the first round of the refinement, we have the previously different ranks leveled.

If we try to use the third power of the corresponding prime of the current rank for this structure then there is no leveling. Generally speaking, this issue arises when we have two or more neighbors of the atom with the same rank. Since in the molecular graphs we never have atom degree higher than 6 (structures like SF_6_), we propose to use the eighth power of the corresponding prime of the current rank to guarantee that leveling or swapping the ranks in the structure never occurs at the refinement step (since 8 = 2^3^, the eighth power can be calculated quickly). Another approach, for which one of the reviewers of this article pointed out, is using the comparator to track the previous rank and current rank which allows to split ties only within each cell of the partition. Such a comparator is also sufficient to prevent ranks swapping at the refinement step.

**Fig.6:**
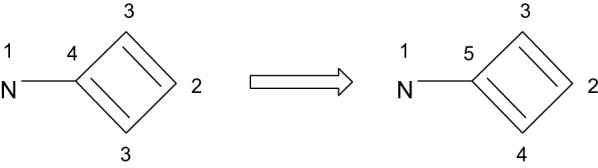
Incorrect (left) and correct (right) symmetry classes for the example of annulene

Another issue with the original CANON algorithm is that it does not take into account the bond orders between the atoms. For example, it determined only four symmetry classes for the structure in Fig. 6 while there are five.

This fact has led to arbitrary choosing between the atoms and generation of the different SMILES by the traversal of the molecular graph. The common workaround of this problem is to choose the bond at the branch point when generating the SMILES, for example, O'Boyle proposes [4]: "Rule C: At each branch point, multiple bonds are favoured over single or aromatic bonds, and lower canonical labels over higher". Such a solution lets to avoid ambiguity of the generation of SMILES but does not solve the issue of the incorrect symmetry perception and this fact can lead to the problems during the canonical traversal of other parts of the molecular graph. We propose to solve this issue by multiplying the eighth power of the corresponding prime of the current rank with the corresponding primes of the ranks of its neighbors in the powers equal to the orders of the bonds connecting the current atom with its neighbors.

Pseudocode of the Modified CANON algorithm is following:

- **algorithm** canon **is**
- **input**: arrays of atoms and bonds of the molecular graph with the initial ranks
- **output**: arrays of atoms and bonds of the molecular graph with the refined ranks
- # Ranking for obtaining the consecutive small integer ranks
- **rank (atoms)**
- dist: = maximal rank of the atoms in the molecular graph
- prevdist: = dist—1
- # while the maximal rank of the atoms is lesser than the count of the atoms and the current maximal rank is # larger than the previous maximal rank perform the refinement
- **while** dist < count of the atoms and dist > prevdist **loop**
- # save previous ranks of the atoms and previous maximal rank of the atoms in the molecular graph
- previous_ranks: = ranks
- prevdist: = dist
- **foreach** atoms **loop**
- rank: = (prime (rank))^8
- **foreach** bonds of current atom **loop**
- rank: = rank * (prime (previous rank of the neighbor atom))^(order of the bond connecting the neighbor atom with current atom)
- **end loop**
- **end loop**
- # Ranking for obtaining the consecutive small integer ranks
- **rank (atoms)**
- dist: = maximal rank of the atoms in the molecular graph
- **end loop**
- **return** atoms with refined ranks

The Modified CANON algorithm terminates when the count of the different values of the ranks ceases to increase from the previous step of the algorithm. After its termination, we must have the same ranks only on the symmetrically equivalent atoms, i.e. the ranks of atoms are their symmetry classes [3]. These symmetry classes can be used for the calculation of the chirality invariant. For acyclic and simple cyclic structures they also can be used directly for further generation of the canonicalized SMILES. If after the use of the Modified CANON algorithm the count of various ranks in the molecular graph is less than the count of atoms, then to obtain unambiguous order of the molecular graph traversal we need to perform breaking ties procedure, as Weininger et al. pointed out [3]. Our method for this is the same as described: doubling all ranks and reducing the value of the atom, which is tied, by one. The set is then treated as a new invariant set, and the previous algorithm for generating an invariant partitioning is repeated. But Weininger et al. assumption that “the double-and-tie-break step does not introduce ambiguity into the ordering since only otherwise equivalent atoms will be tied at any point” can be wrong in the general case. Ivanciuc pointed out [1]: “Two vertices from different atom invariant class cannot be automorphic, while two vertices from the same atom invariant class are not necessarily automorphic. Despite numerous efforts, no vertex graph invariant is known which is sufficient to establish the automorphism partitioning, because for certain graphs non-automorphic vertices are partitioned in the same class.” For this reason, we suggest that only a rigorous way to produce canonical ordering for any molecular graph is to generate permutations for all atoms with the same atom invariant class, obtain SMILES for each permutation by traversal and select lexicographically minimal (or maximal) [1]. Our approach to doing this is: after obtaining symmetry classes choose the ring atoms with the maximal tied rank, perform tiebreaking for each of them, refine each partition by the Modified CANON algorithm, and save it. If these new partitions have ring atoms with tied ranks then repeat tiebreaking for each of them as long as the ring atoms with the tied ranks exist in the partitions. For all partitions with the completely partitioned ranks for the ring atoms, generate SMILES by the traversals and select the lexicographically minimal one as the canonical SMILES for this molecular graph.

After establishing the canonical order of the atoms by the Modified CANON algorithm with, possibly, the tiebreaking further procedure to obtain canonical SMILES corresponds to described by Weininger et al. [3] (procedure GENES): the structure is treated as a tree and a SMILES string is generated that corresponds to a depth-first search (DFS) of this tree. The lowest canonically numbered atom is chosen as the starting point. This atom becomes the root of a tree for a subsequent DFS. Branching decisions are making by directs branching toward the lowest labeled atom at the fork in the branch. For the cyclic structures, Weininger et al. have used a two-pass method: on the first pass, the DFS algorithm detects which bonds will become ring bonds (chords) and on the second pass, ring closure indicators (digits) could be appended to the chords node symbols.

**Fig.7:**
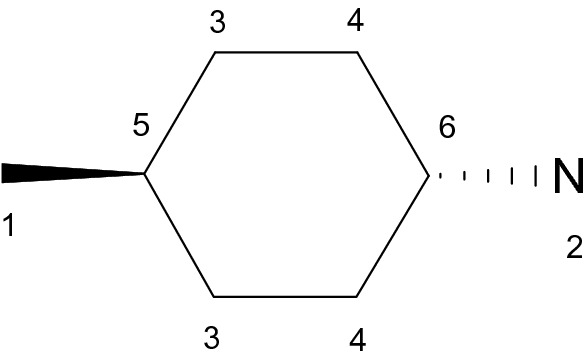
Example of the structure with only dependent chirality and no chiral centers

After the second pass of the DFS algorithm, there is complete information about the canonical order of the atoms in the neighborhood of chiral centers. We use the third pass of the DFS algorithm to obtain canonical SMILES with the correct parity (absolute SMILES): if the canonical order of the atoms on the second pass of the DFS algorithm can be obtained by even number of swaps from the original order of atoms in the molecular graph then the parity symbol (‘@’ or ‘@@’) is preserved. If not then the parity symbol is inverted. We cannot use order relative to the canonical ranks for the correct designation of the chirality because of the structures that have only relative configurations and no chiral centers (structure in Fig. 7 for example).

For such structure, all chirality invariants are equal to 0 and this fact has led to two possible traversals of this molecular graph. Previously described tiebreaking procedure allows us to avoid this ambiguity by the selection of lexicographically minimal SMILES as the canonical one.

Pseudocode of the canonical SMILES generation algorithm is following:

- **algorithm** canonsmi **is**
- **input**: arrays of atoms and bonds of the molecular graph
- **output**: canonical SMILES for this molecular graph
- # determination of the local invariants of the atoms
- **local_invariants (atoms, bonds)**
- # rings detection in the molecular graph and determination of the sizes of the smallest rings for all bonds
- **rings_detection (atoms, bonds)**
- # aromaticity perception and transforming the atoms and bonds to aromatic (if necessary)
- **aromaticity_detection (atoms, bonds)**
- # calculation of the distance invariants for all atoms
- **distance_invariants (atoms, bonds)**
- # symmetry perception (using the local, ring and distance invariants and the Modified CANON algorithm)
- **symmetry_detection (atoms, bonds)**
- # processing of the chiral centers and recalculation of the ranks of atoms (for the chiral structures)
- **chirality_processing (atoms)**
- # generation of all permutation of the atoms ranks by breaking ties on the ring atoms with same ranks
- **while** ring atoms with the tied ranks exist in the partitions **loop**
- **breaking_ties (atoms)**
- # refinement of the partitions after breaking ties by the Modified CANON algorithm
- **canon (atoms, bonds)**
- **end loop**
- # generation of the SMILES for each partition with the completely partitioned ranks
- **foreach** partition **loop**
- # selection of the lexicographically minimal SMILES
- **if cangen (atoms, bonds)** < minsmi **then** minsmi: = **cangen (atoms, bonds)**
- **end loop**
- # return of the lexicographically minimal SMILES as the canonical SMILES for this molecular graph
- **return** minsmi

## Results and discussion

Now, we discuss the computational complexity and the accuracy of the described algorithms.

### Symmetry perception

We have the constant computational complexity for the determination of the local invariants and the chirality invariant of the atoms and the quadratic in a worst-case computational complexity for the determination of the atomic ring invariant: the count of the bonds in the molecular graphs is approximately equal to the count of atoms and we must find the shortest non-trivial path between the atoms incident with each bond by the breadth-first search with the linear complexity in a worst-case. For the same reason, we have the same quadratic in a worst-case computational complexity for the determination of the atomic distance invariants. The Modified CANON algorithm has the same computational complexity as Weininger’s original CANON algorithm [3] and thus we have O(N^2^ log N) general computational complexity for the procedure of the symmetry perception in the molecular graphs.

Use of the atomic ring invariant, the distance invariant and the Modified CANON algorithm allow predicting an accurate count of the vertex equivalence classes for all graphs from the article of Razinger et al. [20] without any explicit determination of the graph automorphisms: for all test graphs in Fig. 6 in this article, we have the same count of the symmetry classes as in Table 1 [20] for HOC and Shelley-Munk algorithms. The correct counts of the vertex equivalence classes are also obtained for all “complex” graphs in Fig. 4 and all regular graphs in Fig. 8 in this article [20], as well for all counterexamples from Carhart’s [9], Figueras [21], Faulon’s [22] and Ouyang’s et al. [23] articles. Due to the explicit use of the bond orders in the main loop of the Modified CANON algorithm, we can correctly determine the count of the symmetry classes in the non-aromatic annulenes and fullerenes (Ivanciuc pointed out [1] to a issues for these classes for the classical Morgan algorithm).

For the determination of the limitations of our algorithm for the symmetry perception, we also tested all 3-regular graphs up to 14 vertices from the article [24] and other cyclic and regular graphs up to the degree 4. The results of testing are presented in the Additional files 1, 2, 3, 4 and 5. We found several examples of the non-planar regular graphs for which our invariants with the refinement by the Modified CANON algorithm cannot determine the accurate count of the symmetry classes such as the generalized Petersen graph G(7,2) (SMILES: C12C3C4C5C1C6C7C2C8C3C6C5C8C74) and 4-regular graphs (graphs with all vertices with the degree equal to 4): the Chvátal graph [25], the Robertson graph [26] and the Brinkmann graph [27] (see the file 3 with 4-Regular Graphs in Supporting Information). This is known that these graphs are not vertex-transitive but our algorithm determines only one symmetry class for each of them. But the non-planar and especially 4-regular graphs usually are not of interest to the chemists since the chemical structures corresponding to these graphs cannot exist due to the sterical constrainings [22]. Also, we found in Table II of the article [10] of Shelley and Munk the example of the molecular graph for which our symmetry perception algorithm incorrectly determines the count of the symmetry classes (SMILES: C1OC23COC45COC11COC67COC8(COC9(CO2)COC(CO1)(CO6)OCC(CO9)(OC4)OCC(CO5)(OC7)OC8)OC3). Our algorithm (as well as the algorithm of Schneider, Sayle, and Landrum [5]) determines for this molecular graph only three equivalence classes of the atoms while, in reality, no vertices are equivalent [10]. These facts once again prove the necessity of the step of the canonical code generation by the automorphism permutation for the robust algorithm of the SMILES canonicalization.

### Generation of the canonical SMILES

The procedure of the graph traversal by the depth-first search has linear computational complexity. But in the case of the symmetrical structures, we must perform the procedure of breaking ties and such count of the graph traversals as the count of the partitions with the completely partitioned ranks for the ring atoms that we get after breaking ties. This count depends on the structure of the molecular graph, but for a great majority of “usual” chemical structures with N non-overlapping bilaterally symmetrical rings, it will be 2^ N^. We prefer to start the breaking ties from the ring atoms with the highest ranks since they have as a rule higher degree and thus tiebreaking on them gives us faster symmetry breaking in the original molecular graph after the refinement procedure. For the complex molecular graphs with overlapping symmetrical rings, the count of traversals is hard to predict, but usually it is not very large, since the refinement by the Modified CANON algorithm after tiebreaking drastically reduces this count. If the algorithm of the symmetry perception determined accurately all symmetry classes of the atoms then the count of traversals equal to the order of the automorphism group of the molecular graph, if not—then the count of traversals will be more than the order of the automorphism group. As examples: for the structure in Fig. 1 (SMILES: C1CC2CCC1CCC3CCC(CC3)CC2) we have 16 traversals, for the structure in Fig. 2 (SMILES: C12C3C1C4C5C4C5C23)—16 traversals too, for the adamantane (SMILES: C1C2CC3CC1CC(C2)C3)—24 traversals, for the cubane (SMILES: C12C3C4C1C5C2C3C45)—48 traversals, for the buckminsterfullerene (C_60_)—120 traversals, for the generalized Petersen graph G(7,2) (SMILES: C12C3C4C5C1C6C7C2C8C3C6C5C8C74) with inaccurate determination of the symmetry—28 travesals. Since the traversals are independent of each other, they can be easily parallelized in the computation. We must say that for almost all structures without chirality investigated by us all generated SMILES are the same for each structure. For this reason, it can seem redundant to generate all traversals, but in very rare cases of the graphs (such as the structure C12C3C4C5C1C6C7C2C8C3C6C5C8C74) this is not true and thus the rigorous way to obtain canonical SMILES is to generate SMILES by all traversals and select the lexicographically minimal SMILES as the canonical one [1]. This is particularly necessary for the structures with only relative (dependent) chirality since all chirality invariants on their atoms are equal to 0 and they can have several equal traversals which led nevertheless to the different SMILES. For example, for structure in Fig. 7 we have two traversals and one of them has led to SMILES C[C@H]1CC[C@H](N)CC1, but another one has resulted in SMILES C[C@@H]1CC[C@@H](N)CC1. We have no other rule of choice between these pair of SMILES except to select the lexicographically minimal SMILES as canonical, i.e. C[C@@H]1CC[C@@H](N)CC1 will be the canonical SMILES for this structure.

It should be emphasized that our procedure of the generation of the canonical SMILES is complete. It contains the graph invariant atom partitioning step (the calculation of the atomic local and nonlocal invariants and refining them by the Modified CANON algorithm for obtaining the symmetry classes of the atoms) and the canonical code generation by the automorphism permutation step (the tiebreaking procedure, refinement of the partitions by the Modified CANON algorithm, and the generation of SMILES by deep-first traversal for each partition). This means that regardless of the accuracy of the symmetry determination, it is mathematically provable that our procedure always gives the same (lexicographically minimal) canonical SMILES for all label permutations of one molecular graph [1]. The only drawback is that in the rare worst cases (molecular graphs with many disjoint symmetrical ring systems) the traversals count could have an exponential dependence on the number of rings in the molecular graph. This fact reflects the computational complexity of the graph automorphism partitioning problem which is well known in the mathematical graph theory for a general case [15].

## Conclusion

We propose new invariant (the product of the corresponding primes for the ring size of each bond of an atom) as a simple unambiguous ring invariant of an atom that allows distinguishing symmetry classes in the highly symmetrical molecular graphs using traditional local and distance atom invariants. Also, we propose modifications of Weininger’s CANON algorithm to avoid its ambiguities (swapping and leveling ranks, incorrect determination of symmetry classes in non-aromatic annulenes, arbitrary selection of atom for breaking ties). The atomic ring invariant and the Modified CANON algorithm allow us to create a rigorous procedure for the generation of canonical SMILES which can be used for accurate and fast structural searching in large chemical databases.

## Supplementary information

**Additional file 1.** PL/SQL source codes of the described algorithms.

**Additional file 2.** Testing results of the symmetry perception algorithm for test graphs.

**Additional file 3.** Testing results of the symmetry perception algorithm for 3-regular graphs.

**Additional file 4.** Testing results of the symmetry perception algorithm for 4-regular graphs.

**Additional file 5.** Testing results of the symmetry perception algorithm for other graphs.

## Data Availability

PL/SQL source codes for the SMILES parsing and the calculation of the local and nonlocal atomic invariants (smilin.txt), the Modified CANON algorithm (canon.txt), the deep-first traversal of the molecular graph (cangen.txt) and the Breaking Ties and the selection of the lexicographically minimal canonical SMILES (canonsmi.txt) are available in archive ChemInf.zip in the supporting information and on the GitHub: https://github.com/Krotko/ChemInf. All types, procedures and functions must be compiled in the order in which they are ordered in the package description file ChemInf.txt. The script smilin.sql is intended for detailed analysis of any molecular graph and establishment of the local and nonlocal invariants of its atoms and bonds. Also the script shows the results of canonical code generation by the automorphism permutation for the complex molecular graph
